# Intraoperative liquid biopsy as a tool for detecting R1 resection during pancreatoduodenectomy in patients with pancreatic carcinoma: the CETUPANC trial (part II)

**DOI:** 10.1097/JS9.0000000000002153

**Published:** 2024-11-15

**Authors:** Javier Padillo-Ruiz, Carlos Garcia, Gonzalo Suarez, Gerardo Blanco, Luis Muñoz-Bellvis, Iago Justo, Maria I. García-Domingo, Fabio Ausania, Elena Muñoz-Forner, Alejandro Serrablo, Elena Martin, Luis Díez, Carmen Cepeda, Luis Marin, Jose Alamo, Carmen Bernal, Sheila Pereira, Francisco Calero, Imán Laga, Sandra Paterna, Esteban Cugat, Constantino Fondevila, Diego López-Guerra, Inmaculada Gallego-Jiménez, Juan José Borrero-Martín, Miguel Ángel Gomez-Bravo, Jose Tinoco, Luis Sabater

**Affiliations:** a Virgen del Rocío University Hospital, IBIS, Seville; b Badajoz University Hospital, University of Extremadura, Badajoz; c University Hospital of Salamanca, Salamanca Biosanitary Institute, University of Salamanca, Salamanca; d University Hospital October 12 in Madrid, Madrid; e Terrassa Mutual University Hospital, Terrassa; f Hospital-Clinic, August Pi i Sunyer Biomedical Research Institute, University of Barcelona, Barcelona; g Valencia Clinical Hospital, University of Valencia, Biomedical Research Institute, Incliva, Valencia; h Miguel Servet University Hospital, Zaragoza; i Princess University Hospital, Madrid; j Clinical Hospital, Madrid, Spain

**Keywords:** CTCs, intraoperative circulating tumor cells, intraoperative liquid biopsy, pancreatic cancer, pancreatic ductal adenocarcinoma, R1 resection

## Abstract

**Introduction:**

A positive surgical margin (R1 resection) is a relevant risk factor for local recurrence in patients with pancreatic ductal adenocarcinoma of the pancreas (PDAC). An intraoperative liquid biopsy (ILB) based on tumor cell mobilization could help to detect R1 resection intraoperatively.

**Objective:**

To evaluate the potential role of the intraoperative circulating tumor cells (CTCs) and cluster mobilization on the R0/R1 detection.

**Methods:**

Sixty-three patients with resectable PDAC of the head of the pancreas were prospective enrolled under the CETUPANC trial. Open pancreaticoduodenectomy (PD) was done in all patients. Intraoperative CTCs and clusters were determined during PD.

**Results:**

The overall rate of R1 resection was 34.9% (22/63 patients). Multivariate analysis showed that factors associated with R1 resection (AUC=0.920) were the presence of undifferentiated G3 tumor (*P*=0.017), microscopic vascular invasion (*P*=0.016), and the intraoperative increase of both free CTCs and clusters in portal vein determination from the beginning to the end of the surgery (*P*=0.002 and *P*=0.005, respectively). A specific logistic regression model, including delta end to baseline CTCs and cluster mobilization to achieve a combined cut-off to detect R1 detection was calculated (AUC=0.799). The obtained R1-index based on ILB had 84% of sensitivity and 68% of specificity to detect R1 resection.

**Conclusions:**

The ILB based on the intraoperative mobilization of CTCs and clusters from the beginning to the end of the PD was a predictive factor to detect R1 resection in patients with PDAC.

## Introduction

Highlights

- Intraoperative liquid biopsy (ILB) based on the intraoperative mobilization of CTCs and clusters from the beginning to the end of PD was a predictive factor for detecting R1 resection in patients with PDAC.
- Analyzed independently, the variation of CTCs and clusters during surgery had high sensitivity for detecting R1 resection and, above all, showed a high negative predictive value for ruling out the persistence of residual tumor.
- Our logistic regression model and the chosen cut-off for surgical margin status showed a negative predictive value of 95% and a sensitivity of 90%, making it highly useful for ruling out a positive margin.

Pancreatic ductal adenocarcinoma (PDAC) is a cancer with a bad prognosis and overall survival (OS) due to the progression of the disease with local recurrence and/or distant metastases after surgery^1,2^.

Currently, surgery done in high-volume centers is considered the main treatment for prolonged survival for patients with localized stage I to II PDAC^3^. In patients with PDAC localized in the head of the pancreas, despite the latest achievements in surgical and perioperative advances in reducing the perioperative mortality, a high recurrence rate continues to limit long-term results after pancreatoduodenectomy (PD)^4^. Unfortunately, even in cases of successful radical resection of PDAC, the rate of recurrence is up to 80% within 5 years after resection^5^.

Several preoperative factors related to the biology of the tumor could influence local recurrence dramatically: high tumor size, tumors with microscopic neural or vascular invasion, or metastatic lymph nodes^6,7^. In addition to this, issues derived from surgical approaches such as positive surgical margins (R1 resection) are also relevant risk factors^8–10^. Recurrence related to the quality of the surgical resection should be differentiated from other recurrences arising from the residual pancreas^10^.

The R0 rate for PDAC is close to 70%^11^. An infiltrated surgical margin has long been associated with bad survival after surgery^12^. Bearing in mind these considerations, additional removal to achieve negative margins may be of great value^13,14^. Current intraoperative detection of R0/R1 resection in these operations remains a challenge.

In order to identify markers for improving diagnosis and prognosis in PDAC, liquid biopsy, including circulating tumor cells (CTCs), cell-free circulating tumor DNA, or circulating tumor extracellular vesicles^15–18^ has been proposed. Intraoperative CTCs and mRNA determinations have been used in several pilot studies to evaluate potential correlation with distant metastases^19,20^.

However, up to now there are no studies that evaluate the potential role of the application of the intraoperative liquid biopsy approach (ILB) for detecting microscopic positive margins during PD in patients with PDAC of the head of the pancreas.

Thus, the aim of the present study was to quantify the intraoperative CTCs and cluster values during PD as an ILB and to determine their potential ability to detect negative or positive tumor margins (R0/R1) after resection of PDAC of the head of the pancreas.

## Methods

### Trial design

The present prospective multicenter study is included under the CETUPANC randomized trial protocol that compares two surgical approaches: no-touch (NT) versus superior mesenteric artery approach (SMA) (NCT 03340844). A group of patients in this study was evaluated in a previous publication on the relationship between a positive ILB and the appearance of early distant metastases, according to the randomized surgical technique^21^. In the present complementary analysis of the CETUPANC trial, patients with PDAC have been included to evaluate the potential role of ILB on R1 resection detection.

The study was conducted at 10 university hospitals that met the quality requirements published by the PD Multicentric Spanish Group^22^. Trial approval was achieved from the Hospital Ethics Committee in accordance with the Declaration of Helsinki (Date: 21st December 2016; Identification number: 1510-M1-17). The study was registered in ClinicalTrials.gov (NCT 03340844) and followed the Consolidated Standards of Reporting Trials (CONSORT) guidelines^22^. Moreover, this work has been reported in line with the strengthening the reporting of cohort, cross-sectional, and case–control studies in surgery (STROCSS) criteria^23^.

### Patients

Recruitment was developed from January 2018 to July 2020. Patients over 18 years of age with resectable PDAC in the head of the pancreas^1,24^ who signed an informed consent form to participate in the prospective study were included. An explicit mention was made regarding the portal vein puncture and the number of punctures in the informed consent. All patients were selected for upfront surgery based on NCCN^24^ criteria, regardless of CA 19.9 level.

Exclusion criteria included patients at high risk for severe disease (ASA IV) according to the American Society of Anesthesiologists^25^, neoadjuvant chemotherapy, liver metastases, or peritoneal cancer detected at surgery when the tumor could not be removed due to arterial invasion, or macroscopic residual tumor.

Sixty-three patients with PDAC who met the preoperative and intraoperative inclusion criteria were included in this study.

### Intraoperative sampling for determining CTCs and clusters

The original protocol included four intraoperative portal vein samples for ILB named from S_0_ to S_3_ according to the published protocol^21^. The first sample (S_0_) was obtained at the beginning of the surgery. This baseline sample was necessary to assess the starting point of the patients and have the reference to compare the possible changes during surgery.

S_1_ was obtained after tumor disconnection of portal vein drainage. This sample sought to know if the tumor mobilization maneuvers prior to disconnecting the tumor drainage to the portal vein generated greater dissemination of tumor cells in the portal vein itself.

S_2_ was obtained after resection. This sample sought to detect possible changes in the number of circulating tumor cells once the PDAC had been resected. With determinations S1 and S2, we attempted to determine possible peaks and valleys during surgery that could impact the prognosis.

Finally, S_3_ was conducted before abdominal closure. This determination at the endpoint of the surgery would allow, on the one hand, to compare with the baseline determination and with the intermediate determination evaluating the CTCs and clusters’ time evolution and, on the other hand, to generate a final liquid biopsy determination with which to analyze possible prognostic impacts.

No complications were associated with the portal vein puncture to obtain ILB samples.

### CTCs isolation, detection, and enumeration protocol

Seven milliliters of whole blood samples were obtained as aforementioned from the portal vein during surgery using direct repeated punctures with a hypodermic needle (25 G ×1 inch). The puncture hole was covered with moist gauze after the blood was drawn to reduce potential bleeding. Once the blood sample was collected, the samples were stored at 4°C during transportation to ensure the sample stability.

All CTCs and cluster determinations were performed at the main center. Peripheral mononuclear blood cells were used to enrich blood samples using gradient centrifugation with Histopaque-1119 and CTCs were isolated using the IsoFlux platform. To perform the specific CTCs enrichment, the Isoflux Epithelial-to-Mesenchymal Transition CTCs Enrichment Kit (EMT Enrichment Kit, Izasa, Catalog N.910-0106) was used. Fluorescent reagents, including anti-CK-fluorescein isothiocyanate (FITC), anti-CD45-indocarbocyanine (Cy3), and Hoechst 33342 were employed for fixing and staining the enriched CTCs. Fluorescence microscopy was used for CTCs detection and enumeration. The Hough transform algorithm was applied for counting CTCs and clusters. This method demonstrated 89% sensitivity and 91% accuracy^18,26^. The CTCs cluster was defined as the presence of at least two or more aggregated CTCs^27^.

### R_0_/R_1_ rate definition and covariates

The pathological study was carried out according to a specific protocol based on Verbeke’s approach^28^ R_0_/R_1_ rates were defined according to established criteria^29^. R1 resection was considered when the distance to the tumor margin was less than 1 mm. Tumor grade differentiation (G1-G3) and TNM classification of malignant tumors were defined according to the guideline criteria^30,31^. Intraoperative determinations such as the duration of surgery, bleeding, or vascular resection were included.

A complementary 3-year follow-up information of the overall cohort including disease-free survival for local recurrence (LRDFS) was included. LRDFS was defined as the time between surgery and the appearance of local recurrence during the 3 years of follow-up. To detect local recurrence, computed tomography (CT-scan) at 3, 6, 9, 12, 18, 24, and 36 months were scheduled. An additional CT-scan was also performed according to the clinical decision.

### Outcomes

The main primary outcome was to evaluate the rate of R1 resection according to the intraoperative number of CTCs (cells/ml) and clusters (clusters/ml) mobilization. Secondary measurements associated with the potential risk of R1 resection included preoperative clinical data and analytic measurements, tumor characteristics, and intraoperative information. An additional outcome was to evaluate the LRDFS according to the R0/R1 rates.

### Statistical analysis

According to the amendment of the CETUPANC protocol dated 2 June 2017, the sample size was carried out to evaluate CTCs mobilization in a specific way in PDAC, unlike the previous sample size that was estimated considering other tumor types.

The sample size was calculated for the main outcome of the trial is to achieve differences in the mobilization of tumor cell markers between evaluated surgical groups included in the CETUPANC protocol (NT vs. SMA). Accepting an alpha risk of 0.05 and a beta risk of 0.1 in a two-sided test, at least 27 subjects were necessary in each surgical group to find a statistically significant proportion difference of tumor cell mobilization expected to be 26%. A dropout rate of 15% to follow-up was anticipated. Therefore, at least 62 PDAC patients needed to be enrolled in the trial.

Due to the lack of studies that evaluate the potential role of the ILB in R1 resection detection, no specific sample size calculation was carried out for R1 detection. According to the obtained data, a power analysis of the collected sample was carried out in order to determine if it was suitable for evaluating the differences between the mean ∆ end-to-baseline CTCs of patients with R0 and R1 resection. According to the means and SD of each group, for a two-sided test, the power of the sample was 72%, which was enough to achieve significant differences.

The cohort characteristics were compared using logistic regression univariate analysis.

The intraoperative samples were evaluated not only independently (S_0,_ S_1,_ S_2_) but also as delta (∆) referred to the baseline: S_1_- S_0_ (∆ resection-to-baseline), and S_2_- S_0_ (∆ end-to-baseline). A more positive difference in ∆ values indicated higher CTCs and/or cluster dissemination at each sampling time.

Factors determining R1 resection were evaluated with both univariate and logistic regression for multivariate analysis. Variables with *P*-values <0.2 at univariate analysis and those considered clinically relevant were considered for multivariate analysis. The best predictive model was chosen according to: the area under the curve of the model (AUC), Akaike Information Criterion (AIC), Bayesian Information Criterion (BIC), and Hosmer–Lemeshow test.

An additional logistic regression model for surgical margin status prediction was calculated considering only ILB results. The best cut-off of this model was chosen according to the ROC curve. Sensitivity, specificity, positive predictive value (PPV), and negative predictive value (NPV) were calculated for this value.

All tests were two-sided, considering a significance level of α=.05, and were analyzed using Stata BE 17.

## Results

### R0-R1 analysis

The overall rate of R1 resection after PD in PDAC patients was 34.9% (22/63 patients). There were no significant differences in the R1 resection rate between surgical techniques: 11 (38%) in NT versus 11 (32.4%) in the SMA approach.

Regarding the R1-affected margin, 18 (28.5%) had the arterial margin infiltrated, 15 (23.8%) the venous margin, and 1 (1.5%) the pancreatic margin. Ten patients (15,8%) had both venous and arterial margins affected. No patients had a positive biliary margin. There were no differences in arterial margin (*P*=0.866) nor venous margin (*P*=0.815) involvement by surgical technique.

Table 1 shows the baseline, intraoperative, and postoperative comparison of R0 versus R1 resection. The main differences between these groups were observed in tumor characteristics. Patients with R1 resection presented higher invasive tumors than R0 resection, with higher vascular, lymphatic, and neural invasion, although they were not statistically significant (*P*=0.36, *P*=0.24, and *P*=0.13, respectively).

**Table 1:** Cohort description.

	Resection status		
Variable	R0 (*n*=41)	R1 (*n*=22)	*P*
Baseline parameters			
Sex *N* (%): Female/Male	19 (46.3) / 22 (53.7)	12 (54.5) / 10 (45.5)	0.24
Age [years], Median (IQR^a^)	66.1 (58.1–75.1)	62.6 (52.8–71.5)	0.23
Diabetes mellitus *N* (%)	10 (24.4)	3 (13.6)	0.42
Arterial hypertension *N* (%)	19 (46.3)	9 (40.9)	0.39
Dyslipidaemia *N* (%)	19 (46.3)	6 (27.3)	0.28
CA 19-9^b^ [U/ml] Median (IQR)	144 (44–422)	267 (51–507)	0.63
Surgical parameters			
Surgery time [min], Median (IQR)	300 (270–360)	300 (270–310)	0.32
Vein resection presence *N* (%)	10 (24.4)	8 (36.4)	0.71
Blood transfusion presence *N* (%)	9 (22.0)	3 (13.6)	0.54
Postoperative pathologist evaluation			
Tumor size, median (IQR)	3.0 (2.0–3.5)	3.1 (2.5–3.5)	0.67
Tumor Stage *N* (%)			
I	6 (14.6)	0 (0)	0.68
II	19 (46.3)	13 (59.1)	
III	16 (39.0)	9 (40.9)	
Tumor grade *N* (%)			
G1	8 (19.5)	5 (22.7)	0.61
G2	24 (58.5)	10 (45.5)	
G3	9 (22.0)	7 (31.8)	
Vascular invasion presence *N* (%)	25 (61.0)	15 (68.2)	0.36
Lymphatic invasion presence *N* (%)	19 (46.3)	13 (59.1)	0.24
Neural invasion presence *N* (%)	32 (78.0)	18 (81.8)	0.13

Comparison of baseline, intraoperative and postoperative parameters according to R0/R1 status.

^a^ IQR, interquartile range.

^b^ CA 19-9, carbohydrate antigen 19-9.

**Figure 1:**
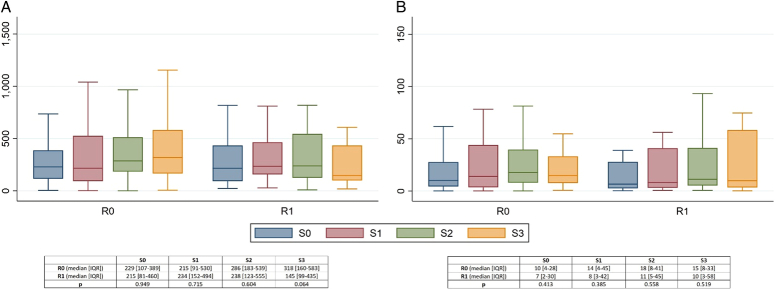
Intraoperative CTCs (A) and clusters (B) time evolution according to R0-R1 status. Boxplots depicting distribution of CTCs and clusters at each intraoperative sample for R0 and R1 resections. A. CTCs. B. Clusters. CTCs, circulating tumor cells; Sampling time from Portal vein: S_0_, initial sampling from the portal vein; S1, after tumor disconnection; S_2_, after tumor resection; S_3_, final surgery.

The comparison of CTCs and cluster evolution throughout surgery according to the R0/R1 resection status is presented in Figure 1. The main peak of both CTCs and clusters was observed after the tumor resection. There were no significant differences between R0 and R1 resections at any sampling time.

A multivariate analysis was performed to determine predicting factors for R1 resection. The model included both parameters related to the tumor characteristics such as the presence of undifferentiated G3 tumor (*P*=0.017) and microscopic vascular invasion (0.016) as well as ILB markers: the intraoperative increase of both CTCs (*P*=0.002) and clusters (*P*=0.005) in portal vein determination from beginning to end of surgery named as ∆ end to baseline. The AUC value of the model was 0.920. The complete result of the logistic regression model is shown in Table 2.

**Table 2:** Logistic regression model for R1 detection in patients with PDAC.

	Univariate		Multiple	
R0/R1 predictor regression	OR (95% CI	*P*	OR (95% CI	*P*
Sex (male)	0.5 (0.2–1.6)	0.241	–	–
Age	1.0 (0.9–1.0)	0.233	–	–
Previous abdominal surgery	0.9 (0.3–2.8)	0.865	–	–
CA 19-9	1.0 (1.0–1.0)	0.625	–	–
Blood transfusion	0.9 (0.2–3.6)	0.881	–	–
Tumor size	1.1 (0.7–1.8)	0.674	–	–
G1–2 vs. G3	5.0 (1.5–17.3)	0.009	19.9 (1.7–230-5)	0.017
Δ Q3-Q0 Free CTC (cell/ml)	1.0 (1.0–1.0)	0.020	1.0 (1.0–1.0)	0.002
Δ Q3-Q0 clusters	1.0 (1.0–1.0)	0.568	1.1 (1.0–1.2)	0.005
Vascular invasion	1.7 (0.5–5.2)	0.362	18.2 (1.7–192.2)	0.016
Lymphatic invasion	1.9 (0.6–5.6)	0.241	–	–
Neural invasion	3.1 (0.6-15.5)	0.134	–	–
Venous resection	1.2 (0.4-4.0)	0.715	–	–
Surgical time	1.0 (1.0–1.0)	0.315	–	–
N+	4.9 (1.1–17.6)	0.018	–	–
Surgical technique	0.9 (0.3–2.7)	0.911		

**Figure 2:**
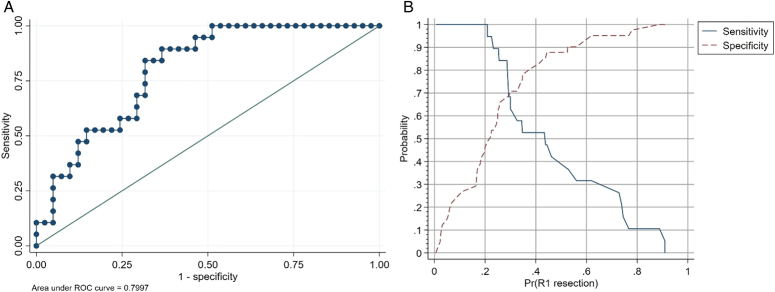
Sensitivity and specificity of ILB including CTCs and clusters for R1 detection. (A) ROC curve of the model with ILB including CTCs and cluster for R1. detection. (B) graph including optimal cut-off points for different combinations of false positive and false negative values, as well as different prevalences of the R1 resection in the population where the predictive model will be employed. According to this graph, the best cut-off for detecting R1 resection was 0.286. This cut-off displayed a sensitivity of 84% and a specificity of 68% (AUC: 0.7997).

In order to assess the accuracy of the ILB in detecting R1 resection by itself, a logistic regression model, including ∆ end to baseline CTCs and cluster mobilization was calculated. The AUC value of the model was 0.799 (Fig. 2). The logistic regression formula for predicting R1 resection during PD resulting from this approach was.

CETUPANC-R1 index: 1/(1+e-(-0.857-0.006×DeltaFreeCTCs+0.047×DeltaClusters)).

The best cut-off to detect R1 resection according to the CETUPANC-R1 index was 0.286. This cut-off has 84% sensitivity and 68% specificity with 55% of positive predicting value and 90% of negative predicting value to detect R1 resection.

#### Local recurrence analysis

**Figure 3:**
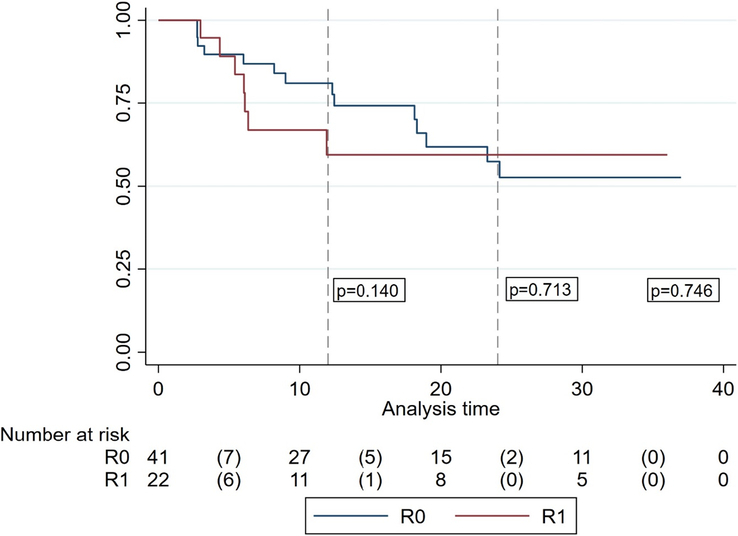
Local recurrence Kaplan–Meier survivor function adjusted by residual tumor classification (*P*=0.854). Dash lines represent highlight recurrences at 12 (*P*=0.140) and 24 months (0.713).

The rate of local recurrence in the cohort was 33.3%. The median time for recurrence (LRDFS) was 6 months (IQR 4–15). Regarding the R0-R1 resection, there were no differences in the rate of local recurrence (32.6% in R0 vs. 35.0% in R1; *P*=0.848) nor in the LRDFS (13 [IQR 6–31] months in R0 vs. 10 [IQR 6–33] months in R1; *P*=0.854). Although it was not statistically significant, a trend towards earlier recurrences could be noticed in the R1 group, with a higher rate of recurrences at 12 months (R0 16% vs. R1 35%, *P*=0.140) (Fig. 3). This trend decreases at 24 months (R0 30% vs. R1 35%, *P*=0.746).

## Discussion

This study describes, for the first time, the potential correlation between CTCs and cluster mobilization during PD and R1 resection in patients with PDAC of the head of the pancreas and ampullary tumors. The ILB based on tumor cell mobilization displayed high sensitivity for detecting R1 resection, although its specificity was lower. These findings could be invaluable for ruling out R1 resection after PD.

Many studies have evaluated the potential role of tumor margins on the prognosis of patients with PDAC and periampullary tumors. Some studies did not find a relationship between positive tumor margins and overall survival, determining that prognosis is mainly associated with vascular invasion, metastatic disease, and factors related to chemotherapy^32,33^. In the present study, early local recurrence at 1 year was higher in R1 resection patients, although differences were not significant, probably due to the limited number of events. However, several retrospective studies^34,35^, clinical trials^36,37^, and meta-analyses^38^ have found that resection margin status is a prognostic factor for overall survival in PDAC. Therefore, achieving a proper oncologic surgical resection with a free margin tumor (R0) is considered the standard of care for PDAC of the head of the pancreas and periampullary tumors^39–41^. However, this goal is not always achieved in PDAC and other periampullary tumors. While the average R0 rate is close to 70% in PDAC, it increases significantly in other indications (90% in ampullary tumors and 80% in distal cholangiocarcinoma)^11^. In the present study, the R0 rate was similar to that described in PDAC (65%), while no affected margins were observed in periampullary tumors. Consistent with other observations, the arterial margin was the most frequently affected.

R1 resection has been associated with tumor characteristics as well as surgical techniques^38^. In this study, multivariate analysis showed that R1 resection was associated with undifferentiated G3 tumors and microscopic vascular invasion. Additionally, intraoperative CTCs and cluster mobilization were predictive factors for microscopic affected margins. This finding suggests that ILB determinations based on circulating tumor cells could be useful for confirming an R0 resection during surgery.

Currently, in order to detect affected margins during surgery, intraoperative frozen section biopsies from resected common bile duct and pancreatic margins are performed prior to complete resection. However, the accuracy of this procedure in PDAC surgery is variable due to histologic heterogeneity related to desmoplastic stromal tissue, chronic pancreatitis, and inflammatory phenomena associated with surgical aggression^42–45^.

Moreover, there is a lack of clear association between the results of intraoperative frozen sections and patient prognosis related to local recurrence and overall survival^43,46,47^. This could be because a complete histopathological evaluation of the whole resected specimen must examine not only bile duct and pancreatic transection margins but also the complete circumferential margin^48,49^. In fact, as observed in the present study, positive margins and local recurrence are mainly observed in the retropancreatic margin as well as in the superior mesenteric artery or vein^13,50^.

Based on the association of intraoperative CTC and cluster mobilization with R1 resection found in the multivariate analysis, a specific study was conducted to evaluate the potential accuracy of the ILB in detecting affected margins after resection by itself. The obtained model allowed us to define an intraoperative index to detect R1 resection based on CTC and cluster mobilization (CETUPANC-R1 index).

Our logistic regression model and the chosen cut-off for surgical margin status showed a high negative predictive value, making it highly useful for ruling out a positive margin. Thus, if the result is negative, extending the surgical margins would not be necessary. However, a positive value would not confirm the involvement of the surgical margin, so the decision of extending the resection^48^ might be made taking into account the clinical suspicion of the surgical team. Although there is still no evidence on the application of hyperthermic chemotherapy^51^ or intraoperative radiation^52,53^ these approaches could be investigated in the future.

To increase the accuracy of our results, the development of a global platform combining circulating tumor cells with other markers such as mRNA or ctDNA has been proposed^54^. This could improve the method’s accuracy for detecting persistent local neoplastic lesions.

The main limitations of the study were the limited number of patients and the isolated use of circulating tumor cells instead of combined use with other liquid biopsy markers that could improve results. Therefore, further investigations are required to enhance accuracy and evaluate the potential role of ILB in detecting R1 resection during surgery. In this sense, we are carrying out a new trial (BLIPANC trial) that includes more patients and other determinations using liquid biopsy.

## Conclusions

In addition to the characteristics of the tumor, ILB based on the intraoperative mobilization of CTCs and clusters from beginning to end of PD was a predictive factor to detect R1 resection in patients with PDAC. Analyzed independently, the variation of CTCs and cluster during surgery had a high sensitivity to detect R1 resection and above all showed a high negative predictive value to rule out the persistence of residual tumor.

## Ethical approval

Approval was obtained from the Hospital Ethics Committee named ‘CI de los Hospitales Universitarios Virgen Macarena y Virgen del Rocío’ in accordance with the Declaration of Helsinki (Date: 21 December 2016; Identification number: 1510- M1-17). The study was registered on ClinicalTrials.gov (NCT03340844) and adhered to the Consolidated Standards of Reporting Trials (CONSORT) guidelines.

## Consent

Written informed consent was obtained from the patient for publication of this case report and accompanying images. A copy of the written consent is available for review by the Editor-in-Chief of this journal on request.

## Source of funding

This work was supported by grants from the Instituto de Salud Carlos III of the Ministry of Health of Spain (PI 16/1465). Main Investigator: Javier Padillo-Ruiz.

## Author contribution

The CETUPANC study is a multicentre randomized clinical performed in 10 University Hospitals. Authors contribution has been: J.P.-R.: concept and design; C.G., J.T., and S.P.: data integrity and accuracy; J.P.-R., C.G., and L.S.: drafting of the manuscript; J.P.-R. and L.S.: critical revision of the manuscript for important intellectual concept; C.G. and J.T.: statistical analysis; F.C. and S.P.: technical support and logistic supervision; J.P.-R.: overall supervision. All authors contributed in acquisition, analysis, or interpretation of data.

## Conflicts of interest disclosure

The authors declares no conflicts of interest.

## Research registration unique identifying number (UIN)

The study was registered on ClinicalTrials.gov (NCT03340844).

## Guarantor

Javier Padillo-Ruiz.

## Data availability statement

Research data supporting this publication are not available from an open repository, although they can be shared by the author in selected cases.

## Provenance and peer review

Not commissioned, externally peer-reviewed.
